# Contextual Effects of Online Review Recency: Three Research Propositions

**DOI:** 10.1007/978-3-030-65785-7_30

**Published:** 2020-11-28

**Authors:** Seunghun Shin, Zheng Xiang

**Affiliations:** 1 Department for Informatics, Technical University of Munich, Garching bei München, Bayern Germany; 2 Smart Tourism Education Platform (STEP) College of Hotel and Tourism Management, Kyung Hee University, Seoul, Korea (Republic of); 3 Department of Tourism and Service Management, MODUL University Vienna, Vienna, Wien Austria; The Howard Feiertag Department of Hospitality and Tourism Management, Pamplin College of Business, Virginia Tech, Blacksburg, VA 24061 USA

**Keywords:** Online review recency, Review helpfulness, Information processing, Contextual factors, Research propositions

## Abstract

Online reviews are influential information sources for tourists in trip planning and related decision-making. How tourists process online reviews is context-specific, so the effects of online reviews on their perceptions or decision-making are affected by different contextual factors. Building on the literature on information recency, this research note discusses recency as an important information component of online reviews and explores a range of contextual factors that affect online review recency in terms of its role in information search and processing. Three propositions are suggested as the basis for future research. Implications for both theory development and managerial practice are also discussed.

## Introduction

Online reviews are influential information sources for supporting tourist’s decision-making [22]. To understand how tourists process online reviews, the literature has studied different information components of online reviews that make them helpful in the eyes of tourists, such as review valence, source credibility, content quality and so on [12, 36]. Review recency, which refers to the degree to which online reviews are current to deliver the latest information about reviewed products or services [34], has been confirmed as a major component [6]. Since a tourism business’s performance may not remain stable over time [15], tourists tend to prefer recent online reviews that indicate its current status of service quality [5, 28].

Review recency manifests in several ways. In general, the posting date of each online review reflects how recently the review was uploaded to the review platform [16]. Along with posting date, some review websites show when the experience occurred to provide further assurance of review recency [15]. For example, in TripAdvisor, each hotel review presents the date of stay along with posting date. Lastly, as online reviews are usually listed in reverse chronological order, their position on the webpage can be an indication of review recency (although only as an approximation) [34].

How tourists process online reviews is context-specific [33]. Essentially, the effects of online reviews on tourist’s perceptions (e.g., review helpfulness) or decision-making (e.g., online hotel booking) become more (or less) pronounced under certain circumstances [11, 13], and so does review recency. For example, while consumers place a high value on recent reviews when they purchase a fast-depreciating product (e.g., fashionable cloth), they do not do so when buying a slow-deprecating product (e.g., classical textbook) [11].

In the hospitality and tourism literature, tourist information processing has been conceptualized as a dynamic process wherein tourists utilize different amounts and types of information sources in accordance with internal and external contingencies [9]. To further understand how tourists process information, it is important to consider the contextual factors which might influence the effects of travel information [7]. However, existing tourism literature has treated the effects of online reviews as static, as if tourist’s online review processing is independent from a range of contextual factors [6, 7].

This research note builds on the literature on information recency to discuss contextual factors that must be considered in order to understand the effects of review recency. Consequently, this study formulates three propositions that guide future research on review recency as well as the dynamic nature of online reviews in the hospitality and tourism field. In this research, contextual factors are defined as the tourist’s (e.g., socio-demographics or travel knowledge), tourism product’s (e.g., product type or popularity), and situational characteristics (e.g., type of device used to process information, time and place of processing information) that may affect the tourist’s information processing.

## Three Propositions on Review Recency

In the hospitality and tourism field, researchers have argued that several different contextual factors affect the way tourists process information [33]. As for the personal characteristics, various socio-demographics have been found as influencing its information search behavior, such as gender [17], age [3], and family life cycle [8]. In addition, other characteristics have been identified as the antecedents of tourist information search, including personality [21] and involvement level [4]. When tourists are highly involved in trip planning, detailed information concerning activities or destinations is carefully considered, but low-involvement tourists tend to disregard the details [4]. Different trip characteristics have been also identified as important contextual factors, such as travel purpose [10], length of travel [24], or travel party composition [15]. According to McKercher [20], while tourists with children tend to focus on the accessibility of restaurants or hotels when they check the information about the properties, couples without children are less concerned about the issue. Lastly, the effects of situational characteristics have been examined. Fodness & Murray [10] have shown that tourists use different information search strategies in response to spatial and temporal dimensions of search activity. In the study of [24], it has been found that tourist’s information search effort is significantly constrained by the available time for the search.

The effects of contextual factors on tourist’s information processing have also been confirmed in the online review setting. Hlee et al. [13] found that the peripheral review components (e.g., the number of reviewers’ followers) become more important criteria for judging review helpfulness, when the review is about unfamiliar restaurants. Findings of the study showed that there are different contextual factors involved in the effects of online reviews on tourist’s perceptions or decision-making. Essentially, it has been argued that the situational nature of the effects of review components should be acknowledged [16].

As such, it is necessary to explore contextual factors that might affect the effect of review recency. Certainly, the contextual effects of information recency on recipient’s perceptions have been examined in other settings. Recency of website content becomes more important for determining its quality when it claims to deliver the latest information (e.g., online news sites) [31]. On online news sites, people select the news story to read based on its recency, but they become more sensitive to news recency when the source of the news is not popular or credible [25, 35]. Based on the literature regarding information recency, we identified three factors associated with online reviews which are expected to influence the effects of review recency in the hospitality and tourism field: context of use, content of online reviews, and nature of the product.

### Context of Use

Although tourists prefer recent reviews to outdated ones in general, their preference for recent reviews could be stronger when they conduct local search (e.g., “restaurants near me”). Tourists search for nearby places of interest the very moment they are needed, so more immediate actions tend to be followed when tourists conduct local search: 76% of consumers who performed local search visited a related business within a day and 88% did so within a week [26]. In other words, tourists deal with spatially close places and make near-future visit decisions in the local search context.

According to the construal level theory (CLT hereafter), when processing information regarding spatially-close objects or near-future events, individuals tend to prefer up-to-date information because it fits their mindset, what is called lower-level construal, and vice versa [29]. Jin et al. [16] found that recent reviews are more influential in affecting consumer’s product choice for near-future purchase decisions. Given this finding and the argument of CLT, it is expected that the effects of review recency on tourist’s decision-making, that is, how influential tourists think recent reviews are as they select the place to visit, become more pronounced when tourists conduct local search.

A recent survey regarding local search revealed that consumers tend to focus on review recency (i.e., how the local business has been evaluated lately) rather than volume (i.e., how popular the local business has been so far) when choosing the place to visit through local search [2]. In the present case, the following research proposition can be formulated.

#### Proposition 1.

* The effects of review recency on tourist’s perceptions or decision-making would be pronounced when tourists use online reviews for local search.*

### Content of Online Reviews

The effects of review recency might be affected by the topics included in its content. As the posting date of each review is readily available, tourists can assess its recency by checking the timestamp [30]. However, the timestamp cannot guarantee that the review includes actual up-to-date content. Even if it has been recently uploaded, the review might not necessarily contain the latest issues related to the subjects [15].

Nowadays, when reading online reviews, tourists want to know how tourism businesses take action on COVID-19 (e.g., measures such as social distance, face covering, hand sanitizer). If the reviews include such information as expected, their recency might be appreciated. However, if there is no information about the safety protocols, even recent reviews would not be perceived as helpful [15].

In present times, social distancing and other actions are considered as important information in restaurant reviews [32]. A new restaurant review website encourages tourists to write about restaurants’ safety protocols rather than other general aspects [23]. In the present case, the following research proposition can be formulated.

#### Proposition 2.

* The effects of review recency on tourist’s perceptions or decision-making would be pronounced when the review content indicates it is up-to-date in addition to its posting date.*

### Nature of the Product

The ability of recent reviews to deliver the latest information is appreciated when their subjects are products whose quality easily changes, such as tourism products [1]. Due to their vulnerability to external factors (e.g., seasonality), the service quality of tourism businesses varies greatly from one time period to another [14]. Thus, tourists want to learn about the current performance of tourism businesses to make better decisions, and they tend to prefer recent reviews to outdated ones [19].

However, even within tourism products, some product’s service quality varies more compared to others. For example, the service quality of restaurants is comparatively less stable than that of hotels [18]. In this case, when processing online reviews about restaurants, tourist’s preference for recent reviews might be pronounced. Conversely, tourists might think that hotel reviews do not need to be up-to-date because the primary aspects of hotel products (e.g., room, staff service, facility) are less time-sensitive [11]. Indeed, it was found that the extent to which tourists assess how recent online reviews are depends on the type of tourism product [27, 37]. In the present case, the following research proposition can be formulated.

#### Proposition 3.

* The effects of review recency on tourist’s perceptions or decision-making would be pronounced when the online reviews concern tourism products whose quality is more time-sensitive.*

## Conclusion

This research note proposes three contextual factors that need to be considered to understand the effects of review recency in the hospitality and tourism context. The propositions can serve as the foundation for future research on online reviews and information recency. Moreover, the current research contributes to the literature on the tourist’s information processing [7, 13] and contextual decision-making, that is, which contextual factors should be considered to provide a more nuanced explanation of how travel information affects the tourist’s decision-making [11, 16]. As for the practical implications, the propositions offer insights into tourism businesses’ online marketing strategies and interface design for information channels. For example, if the effects of recency depend on the product type, then managers can make decisions regarding how frequently they need to check their online reviews or how much effort they should invest in dealing with recent reviews. This could also help local search platforms better understand ways to improve their interface design to enhance the perceptibility of information recency on the result pages in order to meet local search users’ information requirements.
